# ESR Essentials: imaging of suspected child abuse—practice recommendations by the European Society of Paediatric Radiology

**DOI:** 10.1007/s00330-024-11052-4

**Published:** 2024-09-18

**Authors:** Gabrielle C. Colleran, Maria Fossmark, Karen Rosendahl, Maria Argyropoulou, Kshitij Mankad, Amaka C. Offiah

**Affiliations:** 1 Department of Radiology, National Maternity Hospital, Dublin, Ireland; 2 Department of Radiology, University Hospital of North Norway, Tromsø, Norway; 3 Department of Clinical Medicine, UiT The Arctic University of Norway, Tromsø, Norway; 4 Department of Clinical Radiology and Imaging, Medical School, University of Ioannina, Ioannina, Greece; 5 Department of Radiology, Great Ormond Street Hospital, London, UK; 6 Division of Clinical Medicine, University of Sheffield, Sheffield, UK

**Keywords:** Child, Clinical protocols, Diffuse axonal injury, Fractures (bone), Physical abuse

## Abstract

**Abstract:**

The goal of this paper is to provide a useful desktop reference for the imaging of suspected child abuse with clear, age-specific pathways for appropriate evidence-based imaging and follow-up.

We aim to provide a road map for the imaging evaluation and follow-up of this important and vulnerable cohort of patients presenting with signs and symptoms concerning for inflicted injury. As the imaging recommendations differ for children of different ages, we provide a flowchart of the appropriate imaging pathway for infants, toddlers, and older children, which allows ease of selection of which children should undergo skeletal survey, non-contrast computed tomography (CT) brain with 3-dimensional (D) reformats, and magnetic resonance imaging (MRI) of the brain and whole spine. For ease of review, we include a table of the common intracranial and spinal patterns of injury in abusive head trauma. We summarise search patterns, areas of review, and key findings to include in the report.

To exclude skeletal injury, infants and children under 2 years of age should undergo a full skeletal survey in accordance with national guidelines, with a limited follow-up skeletal survey performed 11–14 days later. For children over 2 years of age, the need for skeletal imaging should be decided on a case-by-case basis.

All infants should undergo a non-contrast-enhanced CT brain with 3-D reformats. If this is normal with no abnormal neurology, then no further neuroimaging is required. If this is abnormal, then they should proceed to MRI brain and whole spine within 2–5 days. Children older than 1 year of age who have abnormal neurology and/or findings on skeletal survey that are suggestive of inflicted injury should undergo non-contrast CT brain with 3-D reformats and, depending on the findings, may also require MRI of the brain and whole spine.

We hope that this will be a helpful contribution to the radiology literature, particularly for the general radiologist with low volumes of paediatrics in their practice, supporting them with managing these important cases when they arise in daily practice.

**Key Points:**

- *The choice of initial imaging (skeletal survey and/or brain CT) depends on the age of the child in whom abuse is suspected*.
- *A follow-up skeletal survey is mandatory 11–14 days after the initial survey*.
- *If an MRI of the brain is performed, then an MRI of the whole spine should be performed concurrently*.

## Key recommendations

- In the acute clinical setting of suspected abusive trauma in children under 1 year of age, the appropriate initial imaging protocol is a skeletal survey and an unenhanced brain computed tomography (CT) with 3-dimensional (D) reformats. In children 1–2 years of age, the CT head should be considered on a case-by-case basis. In children 2–5 years of age, both investigations should be considered on a case-by-case basis (level of evidence: low).
- A limited follow-up skeletal survey (routinely including the chest and appendicular skeleton only) after 11–14 days has been shown to increase the sensitivity of initial radiographic imaging and is mandatory where abuse is suspected (level of evidence: low).
- If the head CT is normal, but the child has abnormal neurology and/or a high index of clinical suspicion for physical abuse, brain and whole spine magnetic resonance imaging (MRI) should be performed within 2–5 days (level of evidence: low).

## Introduction

Child abuse is a worldwide occurrence that impacts the well-being of millions across the globe. The youngest children are at the highest risk, with severe physical abuse being 120 times more common in infants compared to children over 5 years of age [1]. The abused child is often non-verbal and may present with diffuse and non-specific symptoms of occult injury, particularly in cases of head and abdominal trauma. Consequently, imaging is central to the diagnosis.

This is a challenging area for the general radiologist, not least because it is known to be an area with serious child and family consequences of over- and under-diagnosis, and high litigation rates. Thus, up-to-date evidence and guidelines are essential to ensure uniformly high-quality care based on currently available literature and to enable confident, competent clinical practice. This article provides concise and clinically relevant practice recommendations for the general radiologist to guide the investigation of a child presenting with suspected physical abuse.

## Craniospinal trauma

### How common is abusive head trauma (AHT) and how is it diagnosed?

AHT is common, with population incidence estimates of 14–53 per 100,000 [2]. AHT is the leading cause of death in infants [3] and toddlers under the age of 2 years [4]. The diagnosis for both accidental and AHT is made from a combination of clinical history and examination by a paediatrician (ideally subspecialising in child abuse), laboratory analysis, and imaging [4].

### How does AHT present?

Presentation is variable, ranging from non-specific clinical features, altered neurological status and seizures, to death (Table 1a).

Developmental delay can be seen in survivors [4]. When the history or proposed mechanism of injury is inconsistent with the clinical examination and severity of findings, a multidisciplinary pathway for assessment for AHT and inflicted injury is triggered.

### Common injury patterns in AHT

**Fig. 1:**
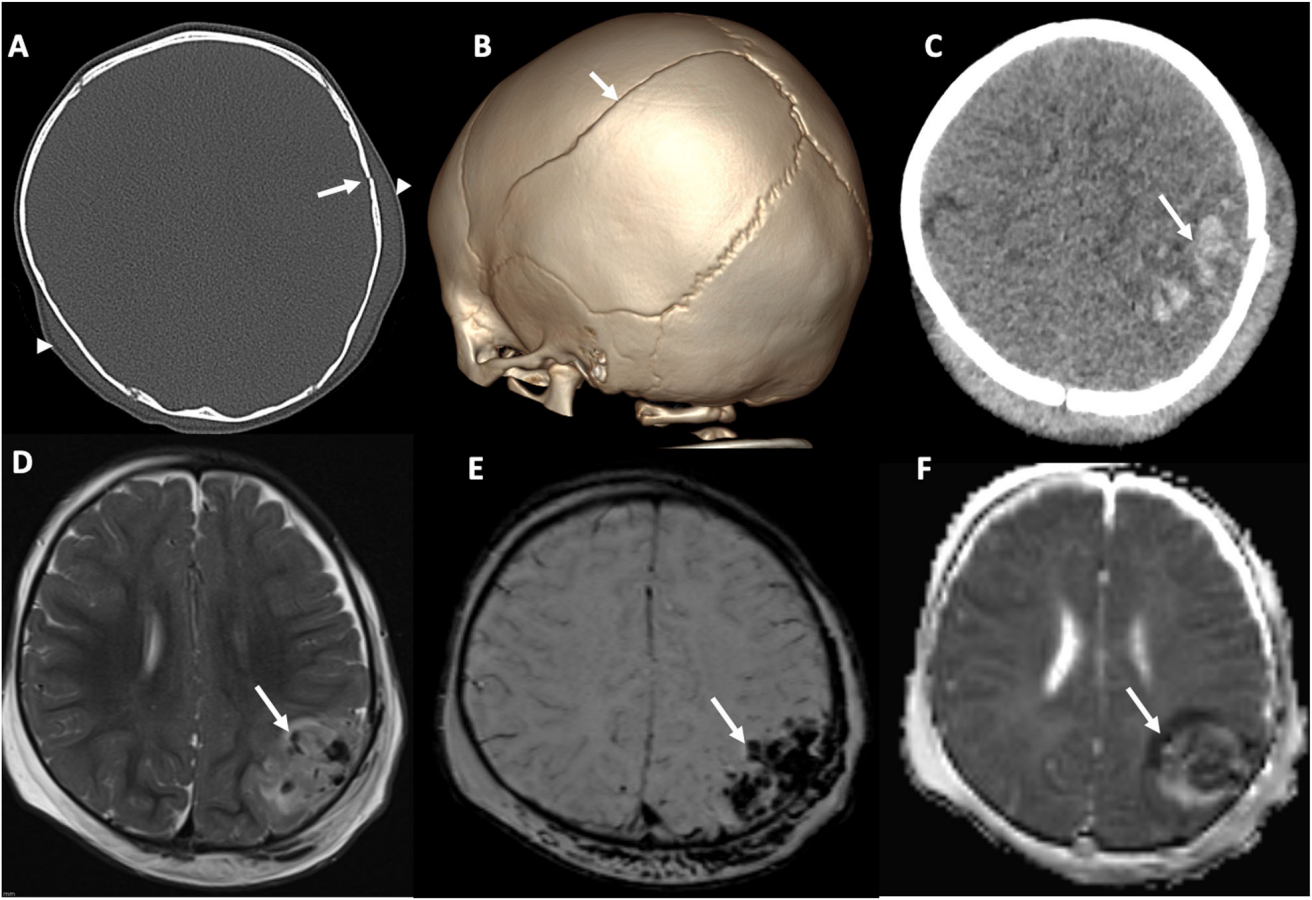
Spectrum of impact injuries. A 12-month-old baby boy presents with a boggy scalp swelling, with no explanation or history of trauma from their parents/carers. **A**, **B** axial CT scan on bone windows (**A**) and 3-D reconstruction (**B**), reveal a slightly displaced left parietal fracture (arrows) extending into the sagittal suture, accompanied by bilateral scalp swelling (arrowheads). **C** Axial unenhanced CT image on parenchymal windows shows an acute, hyperdense left parietal haemorrhagic contusion (arrow). **D**–**F** Axial T2-weighted (**D**), susceptibility-weighted (SWI) (**E**) and parametric apparent diffusion coefficient map (**F**) magnetic resonance images also demonstrate the contusion. Note areas of signal void (arrows) due to susceptibility artefact, more pronounced in the SWI image (**E**)

The common injury patterns in the brain and spine that occur in AHT are outlined in Table 1b and Fig. 1.

**Table 1:** (a) Common presentations of AHT and (b) Common injury patterns that are more specific for abusive than accidental head trauma^a^

(a) Acute	Non-acute	
Irritability	Developmental delay	
Bruises	Enlarging head circumference	
Oral injuries		
Subconjunctival injuries		
Altered level of consciousness		
Seizures		
Coma		
Death		

(b) Intracranial	Calvarium	Spine
Multifocal, bilateral or subdural haemorrhage most common	Complex skull fracture	Spinal subdural
Parenchymal injury most significant		Spinal fracture
Hypoxic ischaemic injury		Craniocervical ligamentous injuries in infants
Cerebral oedema		
Retinal haemorrhages		

^a^ While some findings may be more suggestive of abuse, no individual finding is pathognomonic, and the diagnosis will depend on the combination of all clinical, laboratory and imaging findings

**Fig. 2:**
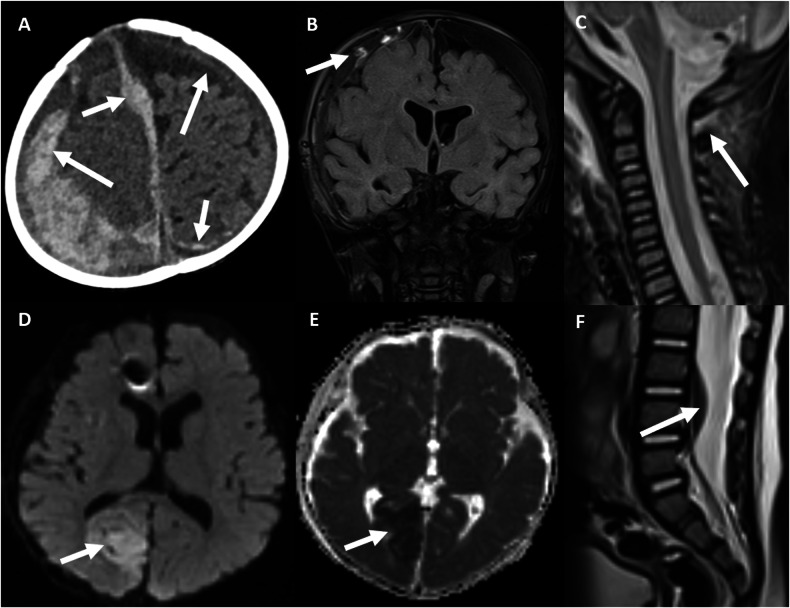
Spectrum of AHT injuries. An 8-month-old baby boy presents in an unexplained floppy and unresponsive state. **A** Axial unenhanced CT scan on parenchymal windows reveals multiple subdural haemorrhagic collections, with mixed density (arrows), one of them being parafalcine (arrowhead). **B**–**D** Coronal fluid-attenuated inversion recovery (**B**), axial diffusion-weighted (DWI) (**C**), and axial parametric apparent diffusion coefficient map (**D**) magnetic resonance images (MRI) of the brain depict subdural collections (arrows) with torn, thrombosed bridging veins (arrowheads in **B**) and hypoxic-ischaemic injury of the right occipital region (arrow in **C**), manifesting with restricted diffusion (arrow in **C**, **D**). A susceptibility artefact in the right frontal area is due to an in situ intracranial pressure monitor (arrowhead in **C**). **E**, **F** Sagittal T2-weighted MRI of the spine reveals cervical spinal ligamentous injury (arrow in **E**) and a spinal SDH (arrow in **F**)

Subdural haemorrhage (SDH) is the most common pattern of intracranial injury with the proposed aetiology being rupture of the bridging veins due to the impulse loading that occurs in the setting of vigorous shaking [5] (Fig. 2). The pattern of SDH varies depending on whether there is accidental or inflicted trauma. Simple/contact impact subdurals are more commonly seen in the setting of accidental injury such as from a fall. Multifocal, bilateral and/or interhemispheric SDH are more associated with AHT/inflicted injury [6]. Extradural haemorrhages are more commonly seen with accidental trauma/contact/impact injuries in infants and toddlers [7].

Complex, bilateral, stellate, or branching fractures are more frequent with AHT than accidental head trauma. However, due to a high incidence of skull fractures from accidental infant and toddler falls, they have a low positive predictive value for abuse.

The identification on MRI of hypoxic-ischaemic injury and cerebral oedema or retinal haemorrhages increases the specificity for AHT [5, 8]. MRI factors that favour an acute SDH include sedimentation of high-density blood products and identification of high-density haemorrhage [5]. Dating of AHT should not be estimated based only on CT density or MRI intensity, but should be based on the entirety of clinical and cranial imaging findings [9].

When assessing the spine, in addition to searching for fractures and spinal SDHs, it is important to pay close attention to the craniocervical junction, as infants are very vulnerable to ligamentous injuries from shaking in this region and these injuries are more common in AHT than in accidental injury [5].

Review areas: (1) Parietal skull, as this is the most common site for skull fracture in AHT. (2) Parafalcine region, as SDH is more common in AHT than in accidental trauma [4]. (3) Bridging veins, as the venous injury is commonly seen in AHT, especially at the junction of the bridging veins with the superior sagittal sinus may be the source of an associated SDH if present [4].

### What do the reports need to include?

The CT report should comment on the presence or absence of skull fracture, scalp/brain parenchymal swelling, SDH (mixed or uniform density), subarachnoid/subpial/epidural haemorrhage, and focal brain parenchymal lesions. Also, document the pertinent negatives. Dating of SDH on CT is complex and should be avoided on the initial CT [4].

**Fig. 3:**
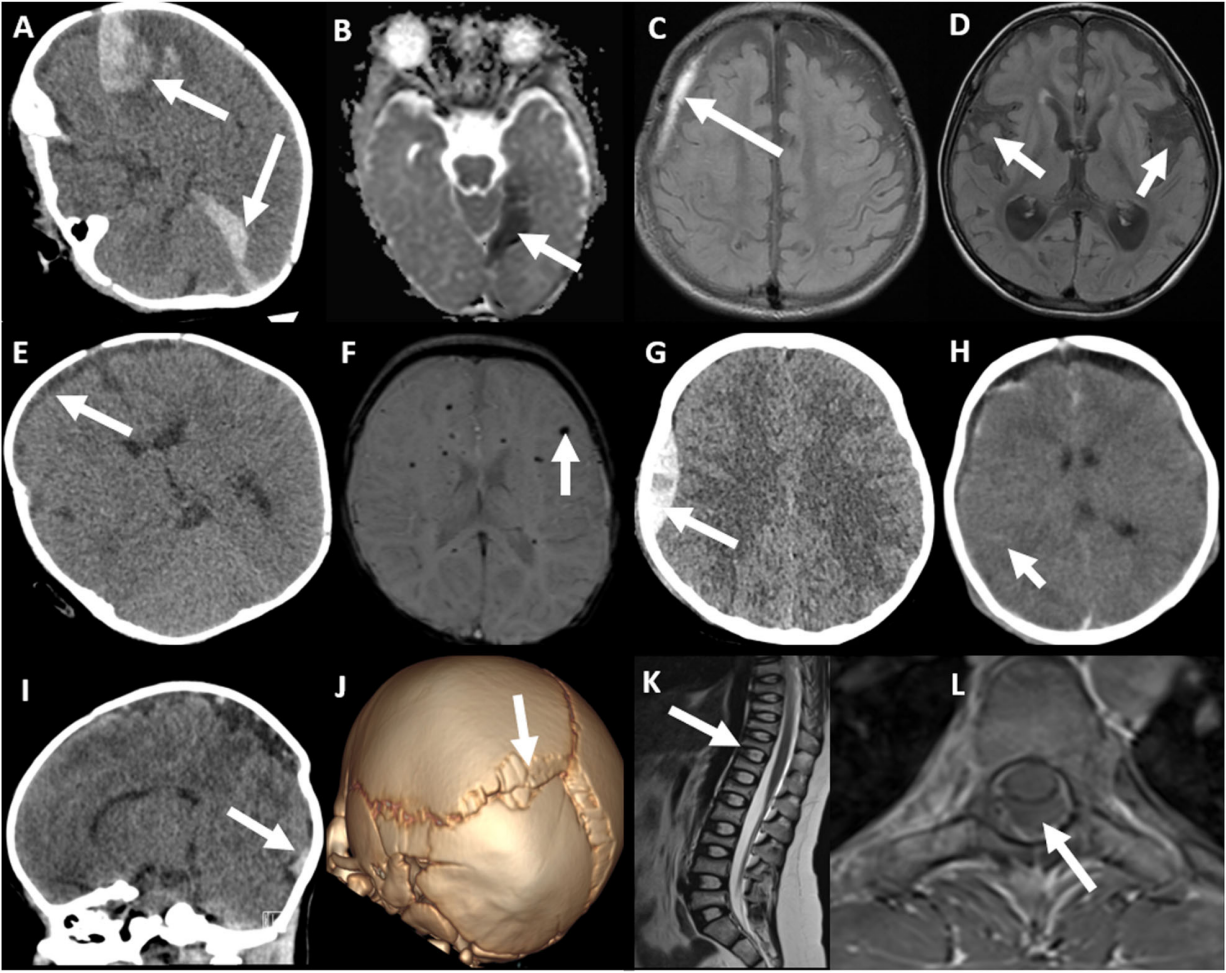
Mimics of abusive craniospinal trauma. All patients shown are infants. **A**, **B** Axial unenhanced CT on parenchymal windows (**A**) and axial magnetic resonance parametric apparent diffusion coefficient map (**B**) images in an encephalopathic girl with factor VIII deficiency show multifocal SDHs (arrows). **C**, **D** Axial fluid-attenuated inversion recovery magnetic resonance images (MRI) in a boy with glutaric aciduria I, show a right subdural collection (arrow in **C**), widened opercula (arrows in **D**), and atrophy of the basal ganglia (arrowhead in **D**). **E**, **F** Axial unenhanced CT image on parenchymal windows (**E**) and susceptibility-weighted (**F**) MRI in a boy with pneumococcal sepsis show small subdural collections (arrows in **E**), and microhemorrhages (arrow in **F**). **G**, **H** Axial unenhanced CT scans on parenchymal windows in a boy (**G**) and girl (**H**) following accidental trauma show a hyperacute extradural haematoma (arrow in **G**) with a skull fracture and SDH (arrowhead in **H**), and subarachnoid haemorrhages (arrows in **H**). **I**, **J** Sagittal reformatted unenhanced CT is presented with a CT scan on parenchymal windows (**I**) and 3-D reconstruction (**J**) in a female neonate with osteogenesis imperfecta showing a birth-related subdural haematoma (arrow in **I**). The 3-D reconstruction displays sutural deformity presenting as a posterior parietal depression and multiple Wormian bones (arrow in **J**). **K**, **L** Sagittal T2-weighted (**K**) and axial T1-weighted (**L**) MRI of the spine in a girl with osteogenesis imperfecta illustrate multilevel vertebral body compression fractures (arrow in **K**), and a post-lumbar puncture spinal extra-axial collection (arrow in **L**)

If the imaging findings raise concern for physical abuse and/or AHT, then a conversation must be had with the referring physicians to ensure the physical abuse protocol skeletal survey and neuroimaging pathway are followed (Fig. 3). The sensitivity of cranial ultrasound is inadequate for clinical use in the setting of suspected AHT and thus is not included in our flowchart [10].

Referral to the child abuse team for assessment is essential and discussion of the findings should be documented in the final report with the name, date, and time of discussion noted [11].

**Fig. 4:**
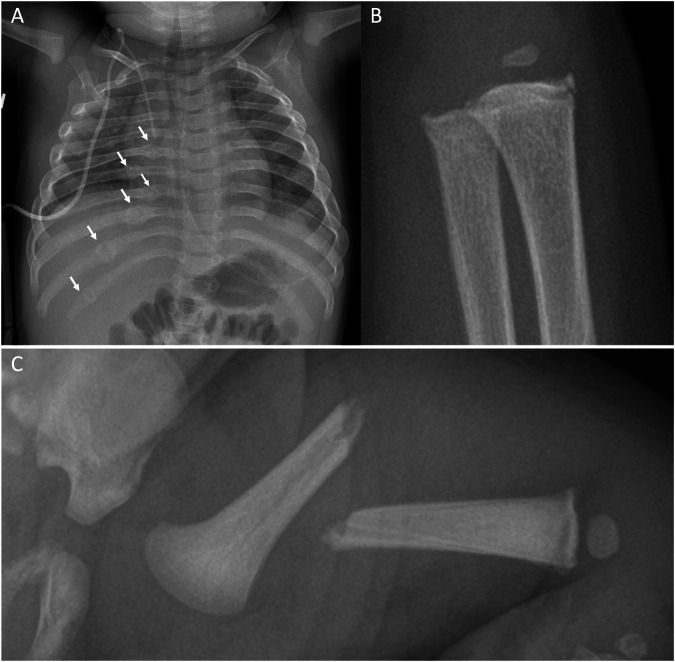
Fractures/presentations typical of inflicted injury. **A** Anteroposterior (AP) chest radiograph in a 9-month-old girl with no history of trauma, shows healing fractures of the posterior arcs of the right 6th–11th ribs (arrows). **B** AP radiograph of the left wrist in an 8-month-old boy shows classic metaphyseal fractures of the distal radius and ulna (circles). **C** AP radiograph of the left femur in a 5-week-old boy shows a displaced and angulated mid-shaft fracture. There is no subperiosteal new bone formation nor is there a callus, consistent with an acute fracture. There was no convincing history of trauma

The MRI report should document the imaging findings including the pertinent negatives and address the presence or absence of intracranial haemorrhage, specifically SDH in the brain and spine, focal or diffuse hypoxic-ischaemic injury, parenchymal injury in the form of contusions, clefts and microhemorrhages, bridging vein thromboses, spinal ligamentous injury, vertebral body injuries, and retinal haemorrhages. Potential mimics of AHT imaging findings are shown in Fig. 4.

If there are specific findings that demonstrate acute or chronic haemorrhage then these should be summarised in the conclusion.

The radiological screening of contact children in the context of suspected physical abuse in the family merits some discussion. The exact incidence is unclear as the literature is sparse. A recent consensus-based guideline was published to address this practice gap, but it awaits further audit-based evaluation [12].

## Skeletal injuries

Fractures in otherwise healthy children under the age of 24 months are rare, with an annual incidence of 0.07% in infants and 0.7% in children aged 12–24 months [9]. In infants, fractures to the femur and tibia predominate as opposed to the tibia and forearm in children aged 12–24 months [13]. Fractures as a result of physical child abuse are significantly more common in children under 18 months of age compared to older children [14]. Up to a third of physically abused children are diagnosed with fractures, many of which are multiple and occult. Any bone may be involved, but some locations have a higher specificity for physical child abuse, such as rib [14] and metaphyseal fractures [15], whereas long bone and skull fractures are the most frequent [16].

### Rib fractures

**Fig. 5:**
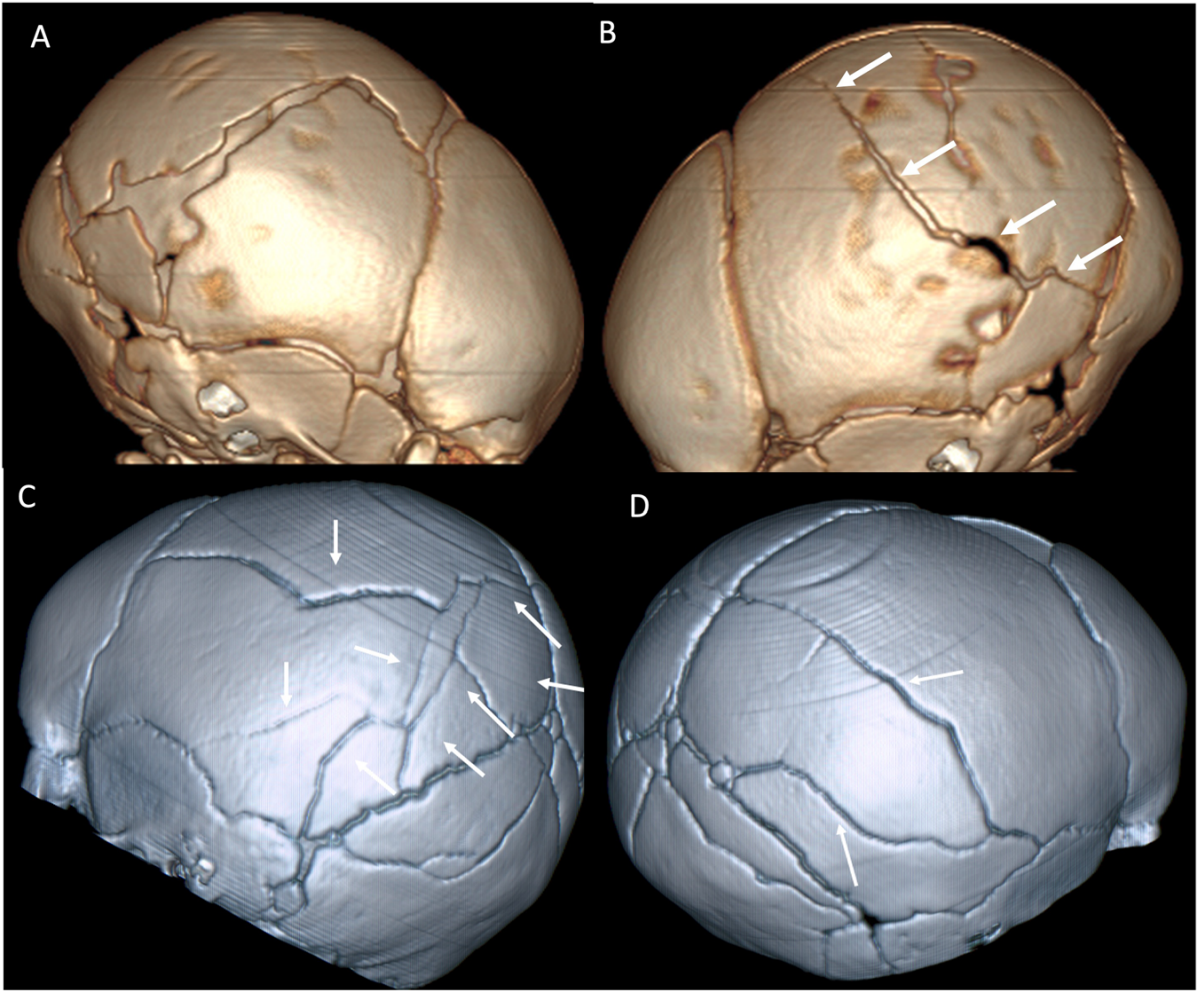
Skull fractures on 3-D reconstructed CT images. **A**, **B** Complex fractures (arrows) of the right parietal bone (**A**) with a displaced quadrangular-shaped fragment (asterisk in **A**) and linear fracture (arrows) of the left parietal bone (**B**) in a 6-week-old boy. **C**, **D** Complex fractures of the left (**C**) and right (**D**) parietal bones (arrows) in a 5-month-old boy

In children under the age of 3 years, rib fractures are estimated to have a positive predictive value for physical abuse of 95% [17], with reported prevalence of rib fractures in cases of suspected child abuse at 14% [16]. Abusive rib fractures can occur at any point along the rib, from the costovertebral articulations to the costochondral junctions [14] (Fig. 5).

Abusive rib fractures are typically multiple, located in posterior and lateral locations from ribs 5 through 8, and more commonly on the left [18]. In up to 29% of cases, one or more rib fractures are the only skeletal finding in an abused child, underscoring the importance of high-quality radiographs and an eye for detail [17]. Chest CT might be considered in children with indeterminate chest findings on skeletal survey [19].

### Metaphyseal injury

In the appropriate clinical setting, classic metaphyseal fractures (corner/chip or bucket handle fractures) are considered the most specific radiographic injury of physical abuse [15]. They are most frequently seen in the distal femur, the tibia, and the proximal humerus, but also occur in the elbow, wrist (Fig. 5), and shoulder [15]. Small undisplaced classic metaphyseal fractures tend to heal within 4 weeks without callus formation by gradual bone consolidation, whereas healing of larger or displaced fractures may take 6–8 weeks. When the adjacent periosteum is injured, subperiosteal haemorrhage occurs, which, during the healing process, is evident as a periosteal reaction. Classic metaphyseal fractures must be differentiated from normal growth variations, which may take the form of subtle irregularities, ‘step-off’ (also termed a metaphyseal collar) or even mimic a small avulsion [20]. In children over the age of 12–15 months with genu vara, metaphyseal fragmentation can be seen due to abnormal stresses associated with early weight bearing [21].

### Long bone fractures

In cases of suspected child abuse, long bone fractures have been documented in 26% of infants. Fractures to the long tubular bones in physically abused children most commonly involve the femur and tibia, followed by the forearm [16] (Fig. 5). Mid-shaft fractures of the humerus in infants are more common in cases of physical abuse than non-abuse [14]. Without a plausible explanation of a significant high-energy impact (e.g. a road traffic accident), discovering a long bone fracture in a pre-ambulant infant should always raise suspicion of physical abuse. Physiological periosteal new bone formation along the shaft of the long bones is a common feature in infants between the ages of 1–4 months of age, and should be differentiated from isolated subperiosteal new bone formation [20].

### Skull fractures

Skull fractures co-occurring with intracranial injury are significantly associated with physical child abuse, in contrast to isolated skull fractures, where there is no such association [22]. Skull fractures have been reported in 24% of infants with suspected child abuse [16] and typically involve the parietal bone.

**Fig. 6:**
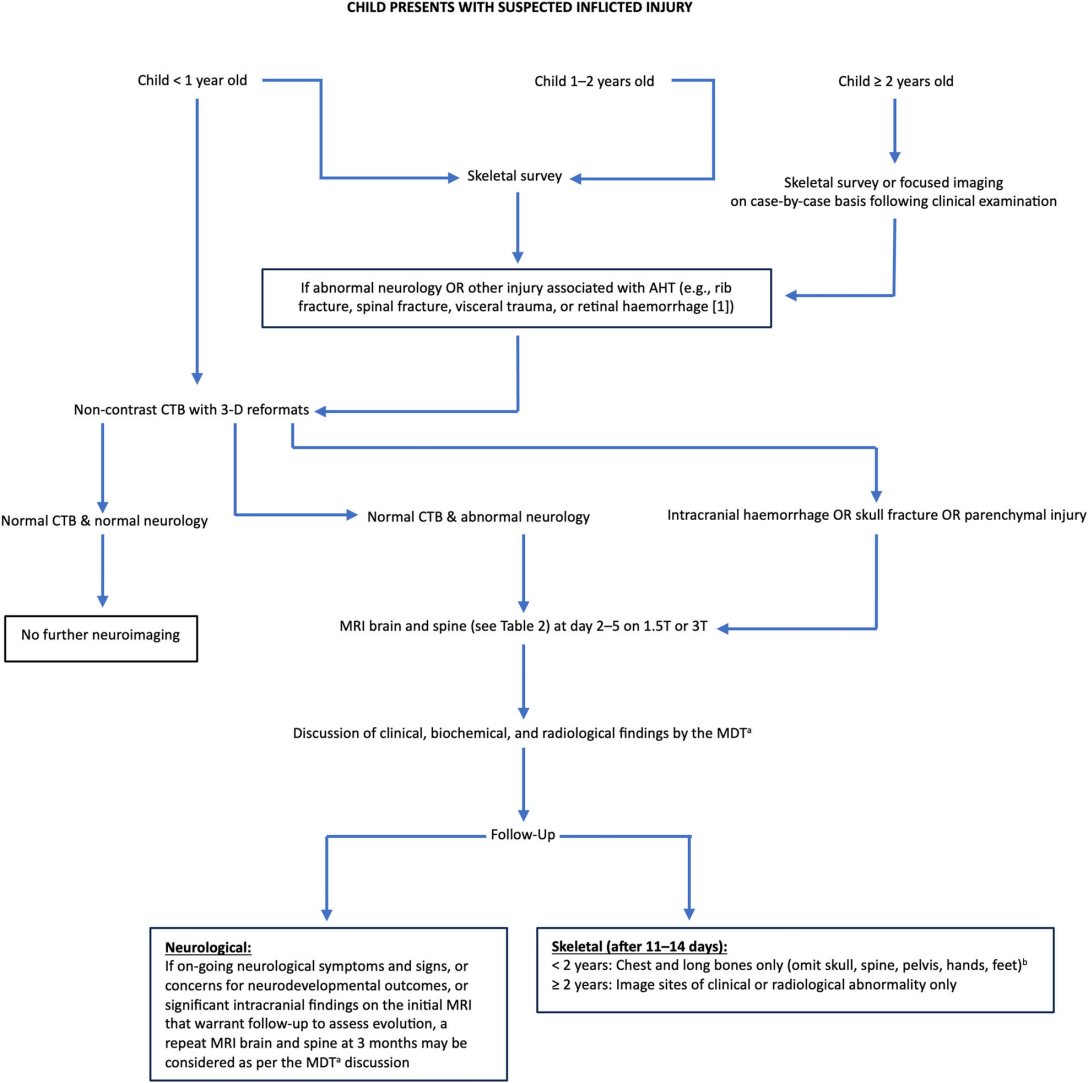
Suspected child physical abuse investigative pathway. ^a^If the local multidisciplinary team is not able to reach a decision, referral to a specialist centre is recommended. ^b^Additional sites (e.g. hands/feet) may be imaged depending on findings on the initial survey. AHT, abusive head trauma; CTB, computed tomography brain; D, dimensional; MDT, multidisciplinary team; MRI, magnetic resonance imaging, T, tesla

Most fractures are linear [14, 16]. In infants examined with a skeletal survey due to suspicion of physical abuse, 19% of patients with a simple skull fracture had a positive skeletal survey, whereas 27% of infants with complex skull fractures had a positive skeletal survey [16]. Irrespective of whether accidental or due to AHT, parietal skull fractures are the most common skull fracture [5]. Complex, bilateral, stellate, branching fractures crossing suture lines favour an abusive mechanism (Fig. 6).

### Unusual fractures

Fractures to the spine, scapula (most commonly the acromion), sternum, pelvis, fingers, and toes, in a pre-ambulant child are rare and should raise concern when no plausible explanation can be provided [16].

### Fracture healing

It is impossible to exactly date a fracture, however, rib and long bone fractures in young children may be classified as acute (< 1 week), recent (8–35 days), or old (≥ 36 days) on the basis of six key radiological features; soft-tissue swelling, periosteal reaction, soft callus, hard callus, bridging, and remodelling [23].

### Differential diagnosis

The differential diagnoses of fractures that may be a result of physical child abuse include accidental injury, including birth trauma, and generalised bone disease. Fractures due to birth trauma most commonly involve the clavicle, femur, or humerus. Differential considerations for bone injury include metabolic disorders such as rickets, copper deficiency (particularly Menkes syndrome), metaphyseal chondrodysplasia type Schmid, spondylometaphyseal dysplasia, and osteogenesis imperfecta [24].

## Visceral injuries

Visceral injuries as a result of physical child abuse are uncommon and may be difficult to identify clinically [25], nevertheless, they constitute a significant cause of mortality and morbidity in affected children. Any organ can be involved, but hepatic and bowel injuries are the most common, while injuries to solid and hollow organs are equally common [26]. Abusive abdominal injuries are mostly seen in toddlers, whereas accidental abdominal injuries are more prevalent in older children. When abusive abdominal injury is suspected, the recommended radiological imaging modality is contrast-enhanced abdominal and pelvic CT, as it is in cases of suspected accidental abdominal injury [26].

## What is the appropriate imaging pathway in cases of suspected physical abuse in children?

Figure 6 outlines the appropriate initial and follow-up investigations for children with suspected physical abuse. The investigations depend on the age of the child, the presence or absence of abnormal neurology on examination and the presence or absence of other physical injuries.

### Imaging of the neuroaxis

Children under 1 year with suspected AHT should all have a high-resolution non-contrast CT with 3-D reformats [11]. If this is abnormal (intracranial haemorrhage, skull fracture, parenchymal injury), the infant should proceed to an MRI brain and spine within 2–5 days. If this is normal, with no abnormal neurology and no suspicion of a non-accidental form of injury, then no further imaging is required. If, however, the CT is normal but there is abnormal neurology, or a high index of clinical suspicion for physical abuse, then an MRI brain and whole spine should be performed within 2–5 days. When an initial MRI brain is performed for suspected AHT, an MRI of the whole spine should always be performed at the same time, due to the high likelihood of associated spinal findings. MRI brain and spine has increased sensitivity for parenchymal injury, intracranial haemorrhage, and fluid collections [5], and allows further delineation of extra-axial collections, assessment of septations within collections, and differentiation of acute from chronic SDHs. Dating of extra-axial haemorrhage is notoriously challenging, and is a topic of much debate in the court setting because of the impact of factors such as admixture of blood and cerebrospinal fluid (when the arachnoid is torn) and clotting status on the appearance of SDH [5]. Susceptibility-weighted imaging (SWI) is useful for the detection of micro and macro haemorrhages. Short tau inversion recovery (STIR) sequences increase the sensitivity of the MRI spine for acute fractures and ligamentous injuries [5]. Protocols for MRI brain and spine for the various age groups are provided in Tables 2 and 3. Asymptomatic contact children younger than 1 year should have neuroimaging, the preferred modality for which is MRI [12].

**Table 2:** (a) Parameters for neonatal brain MRI for suspected AHT, (b) parameters for brain MRI for suspected AHT in infants 4–6 months old, and (c) parameters for brain MRI for suspected AHT in infants 6 months old and above

Sequence	FOV in cm	Slice thickness	Slice spacing
(a)			
Ax T2 propeller	20	3	0.3
Ax T1 propeller	20	3	0.3
Sag T1 propeller	20	3	0.3
DWI propeller + ADC	22	4	0.5
T1 volume	24	1	No slab wrap: 1.06
(b)			
Ax T2 FSE	22	3	0.3
Sag T1 FLAIR	22	3	0
Ax T1 FLAIR	22	3	0
AX DWI MUSE b0-1000 + ADC	22	3	0.3
T1 volume	24	1	No slab wrap: 1.06
SWI (volume)	22	1	No slab wrap: 1.04
(c)			
Ax T2 FSE	24	3	0.3
Ax T2 FLAIR FS	24	3	0.3
T1 volume	25.6	0.5	No slab wrap 1.03
Ax DWI MUSE b1000 + ADC	24	3	0.3
SWI (volume)	22	1	No slab wrap 1.04

*ADC* apparent diffusion coefficient, *Ax* axial, *DWI* diffusion-weighted imaging, *FOV* field of view, *Sag* sagittal, propeller (GE) equivalent to BLADE (Siemens) and MultiVane (Philips), *FLAIR* fluid-attenuated inversion recovery, *FSE* fast spin echo (GE) equivalent to turbo spin echo (TSE) Philips and HASTE (Siemens), *MUSE* multiplexed sensitivity encoding (GE) equivalent to image-space sampling function (IRIS) (Philips) and readout segmentation of long variable echo trains (RESOLVE) Siemens, *SWI* susceptibility-weighted imaging

**Table 3:** (a) Spine protocol for suspected inflicted injury in children < 2-years-old and (b) spine protocol for suspected inflicted injury in children ≥ 2-years-old

Sequence	FOV in cm	Slice thickness	Slice spacing	NEX
(a)				
Sag T1FSE	16	3 mm	0.3 mm	2^a^
Sag T2 FSE	16	3 mm	0.3 mm	2^a^
Ax T1 FSE	16	4 mm	1 mm	2^a^
Ax T2 FSE	16	4 mm	1 mm	2^a^
Ax T2 CC junction	20	1.5 mm	0 mm	3
(b)				
Sag T1FSE	28	3 mm	0.3 mm	2^b^
Sag T2 FSE	28	3 mm	0.3 mm	2^b^
Ax T1 FSE	20	4 mm	1 mm	2^b^
Ax T2 FSE	20	4 mm	1 mm	2^b^
Ax T2 CC junction	20	1.5 mm	0 mm	3
Sag STIR	24	3 mm	0.3 mm	1.5
Sag T1 FLAIR^c^	28	3 mm	0.3 mm	2

*Ax* axial, *CC* craniocervical, *FLAIR* fluid-attenuated inversion recovery, *FOV* field of view, *FSE* fast spin echo (GE) equivalent to turbo spin echo (TSE) Philips and HASTE (Siemens), *NEX* number of excitations, Sag sagittal, *STIR* short tau inversion recovery

^a^ For the lumbar spine, NEX can be reduced to 1.5 as there are fewer sources of artefact in this region (e.g. less impacted by respiration)

^b^ For the lumbar spines, NEX can be reduced to 1.5 as there are fewer sources of artefact in this region (e.g. less impacted by respiration)

^c^ Although not our sequence of choice due to the associated increased specific absorption rate, this is a good option when tissue contrast is not optimal

### Skeletal survey

The role of skeletal imaging in cases of suspected child abuse is to accurately detect (and possibly date) any injuries, to exclude normal variants of growth (which may mimic injuries or fractures), and to diagnose any underlying metabolic or genetic disorders of bone, which may predispose a child to pathological fractures. A skeletal survey is ideally performed semi-electively and during working hours when experienced staff are available, within 24 h and no later than 72 h after the request has been received. The skeletal survey should be reviewed in real-time by the radiologist while the child remains in the radiology suite, so that additional images can be performed if needed [24]. For weekend and overnight presentations, the child may be able to be imaged the next working day or following the weekend, especially if remaining an inpatient. High-quality images of each anatomical site should be performed [27]. A suggested protocol is given in Table 4a.

**Table 4:** (a) Initial skeletal survey protocol for children < 2-years-old [24] and (b) routine follow-up skeletal survey in children < 2-years-old [24, 26]

(a) Region	View	Comment
Skull	AP	Skull is not necessary if CT performed
Skull	Lateral	Skull is not necessary if CT performed
Chest	AP	To include the shoulders and sternum
Chest	Lateral	
Chest	Obliques	Both sides. To include all ribs from 1 to 12
Abdomen and pelvis	AP	
Whole spine	Lateral	
Cervical spine	AP	
Whole arm^a^	AP	Centred at the elbow if possible
Elbow	Lateral	Coned
Wrist	Lateral	Coned
Hand and wrist	Posteroanterior	
Whole lower limb^b^	AP	Hip to ankle
Knees	AP	Coned
Knee	Lateral	Coned
Ankle	Lateral	Coned
Ankle	AP (mortise view)	Coned
Foot	Dorsoplantar	
Any suspected shaft fracture	Lateral	

(b) Region and view	View	Comment
Any abnormal or suspicious areas detected on the initial skeletal survey		
Chest	AP	To include the shoulders and sternum
Chest	Both obliques	To include all ribs from 1 to 12
Whole arm^c^	AP	
Hand and wrist^d^	AP	
Whole lower limb^e^	AP	Hip to ankle

*AP* anteroposterior, *CT* computed tomography

^a^ In larger children where a single whole arm view is not possible: AP humerus (including the shoulder and elbow), AP forearm (including the elbow and wrist), coned lateral elbow, coned lateral wrist, dorsoplantar hand, and wrist

^b^ For larger children: AP femur, AP tibia and fibula, AP knee, AP ankle, coned lateral knee, coned lateral ankle, dorsoplantar foot

^c^ AP humerus including shoulder and elbow, AP forearm (including elbow and wrist) and posteroanterior hand and wrist in larger children

^d^ Dorsoplantar in larger children

^e^ AP femur, AP tibia and fibula in larger children

Anteroposterior (AP) and lateral skull radiographs are part of the standard initial skeletal survey but recent evidence demonstrates that where multiplanar CT with 3-D reformats is performed there is no additional value from the skull radiographs and thus they can be omitted from the protocol [28].

A limited follow-up skeletal survey (omitting the axial skeleton and pelvis) after 11–14 days has been shown to increase the sensitivity of initial radiographic imaging and is mandatory where abuse is suspected (Table 4b) [29].

In other words, the investigation for abuse is not complete until both initial and follow-up skeletal surveys have been performed. Contact children (siblings, cohabiting children, or children under the same care as a child with suspected physical abuse) should undergo a physical examination and a history elicited prior to imaging. Contact children aged 12–24 months should undergo a skeletal survey [12].

### Recommendations

#### Children younger than 1 year of age

- In the acute clinical setting of suspected child abuse, the appropriate imaging protocol is a skeletal survey and an unenhanced CT brain with 3-D reformats [4, 30–34]. If the head CT is normal but there is abnormal neurology, or a high index of clinical suspicion for physical abuse, a brain and whole spine MRI should be performed within 2–5 days, see flowchart. A limited follow-up skeletal survey (omitting the axial skeleton and the pelvis) after 11–14 days should be performed [27].
- Chest CT can be considered in children in this age group with negative skeletal survey and high clinical suspicion for child abuse, and when the radiographic diagnosis of rib fractures is indeterminate [19].
- Children with suspected injury to the abdomen should have a contrast-enhanced CT scan [35].

#### Children 1–2 years of age

- Initial and follow-up skeletal surveys and CT chest and abdomen are performed as for children below 1-year-old (see ‘Children younger than 1 year of age’).
- Neuroimaging is performed as for children above 2-years-old (see ‘Children above 2 years of age’).

#### Children above 2 years of age

- Children above 2-years-old who present with a history of falls or unexplained physical findings such as bruising will usually have a CT brain performed in the acute setting, with the advantage of its exquisite sensitivity for fracture. Where the history is more vague and the symptoms less specific, an MRI within 2–5 days may be the first investigation of the neuroaxis [5]. A skeletal survey is of less value in children above 2, and should be considered on a case-by-case basis. Alternatively, the radiographs may be tailored to the area(s) of suspected injury.
- All children with suspected injury to the chest or abdomen should have a contrast-enhanced CT scan [19, 35].

## Summary statement

Abusive trauma is not uncommon in clinical practice. The radiologist plays an essential role in the diagnostic pathway. A skeletal survey non-contrast CT, and MRI brain and spine are core components of the assessment and should be performed in line with RCR and local guidelines [11]. We have provided a flowchart of imaging sequences and protocols for the skeletal survey and brain and spine MRI, as well as common presenting symptoms and imaging findings. Communication with involved clinicians and documentation of this communication is a core component of the radiological assessment.

## Patient summary

Inflicted injury and abusive head trauma refer to child abuse. This article provides an evidence-based summary of the appropriate imaging tests that children should undergo when there is clinical concern for child abuse.

## Abbreviations

AHT: Abusive head trauma
SDH: Subdural haemorrhage
